# A Spatial Autocorrelation Method for *Taenia solium* Risk Mapping: The Case of Lao PDR

**DOI:** 10.3390/tropicalmed8040221

**Published:** 2023-04-10

**Authors:** Andrew Larkins, Mieghan Bruce, Amanda Ash

**Affiliations:** 1 School of Medical, Molecular and Forensic Sciences, Murdoch University, Perth, WA 6150, Australia; 2 Centre for Biosecurity and One Health, Harry Butler Institute, Murdoch University, Perth, WA 6150, Australia; 3 School of Veterinary Medicine, Murdoch University, Perth, WA 6150, Australia

**Keywords:** Lao PDR, neurocysticercosis, neglected tropical diseases, *Taenia solium*, zoonoses

## Abstract

Background: The World Health Organization has identified *Taenia solium* mapping tools as an important development for intensifying control in hyperendemic areas. *Taenia solium* has also been identified as a priority by the Lao PDR government. There is a limited understanding of the distribution of *T. solium* due to inherent diagnostic challenges. Method: Global and local autocorrelation statistics were applied to available risk factor data sourced from national censuses to map the risk of *Taenia solium* in Lao PDR. Results: Approximately 50% of villages could be considered hot spots for one or more risk factors. Different risk factor hot spots co-occurred in 30% of villages. Twenty per cent of villages were classified as hot spots for the proportion of households owning pigs and another risk factor. Northern Lao PDR was the dominant high-risk area. This is consistent with passive reports, limited surveys, and anecdotal reports. One smaller area in southern Lao PDR was also identified as high-risk. This is of particular interest because *T. solium* has not previously been investigated in this area. Conclusions: The methods applied provide a simple, rapid, and versatile approach that allows endemic countries to begin mapping the risk of *T. solium* at a sub-national level.

## 1. Introduction

*Taenia solium* has been recognised by the World Health Organization (WHO) as the world’s most significant foodborne parasite and the leading cause of preventable epilepsy in low- and middle-income countries [1,2]. It is a zoonotic parasite with a lifecycle predominantly involving humans and pigs. As the definitive host, humans are susceptible to taeniasis, a condition in which adult *T. solium* worms develop and are carried in the gastrointestinal tract after ingesting raw or undercooked pork containing viable cysticerci. People with taeniasis generally do not suffer from substantial illness; however, they distribute infectious eggs or proglottids into the environment via their faeces. These eggs or proglottids are a source of infection for the carriers themselves, other people, and pigs. If ingested, both pigs and humans may become intermediate hosts and develop cysticercosis. Cysticercosis in humans can have major consequences on a person’s health, particularly when cysts develop in the central nervous system, a condition known as neurocysticercosis (NCC). The most common consequence of NCC is epilepsy [3]. Beyond the immediate illness, patients potentially lose the ability to earn an income and participate in daily life, and they may become ostracised by their community [4].

Definitively diagnosing a person with NCC can be a difficult task and requires advanced neuroimaging, brain or spinal cord biopsy, or, in rare cases, visualization of subretinal cysticerci [5]. These diagnostic tools are rarely available in the low and middle-income countries where *T. solium* is endemic. Not only is NCC difficult to definitively diagnose, but methods for diagnosing porcine cysticercosis and human taeniasis due to *T. solium* also present challenges. Often, tests are inaccessible for use in endemic communities, do not differentiate between different *Taenia* species, or suffer from poor field performance [6,7]. An accurate, precise, and field-ready diagnostic test for any stage of *T. solium* does not currently exist, and this is a major limitation to understanding the true extent of the disease in endemic countries. Despite the inherent complexities, foundational observational research has identified multiple key risk factors related to poor water, sanitation, and hygiene; pig rearing; pork consumption; and cultural behaviours [8,9].

### 1.1. Risk Mapping

Given the diagnostic challenges of *T. solium*, the WHO has called for the development of mapping tools that have the potential to identify hyperendemic areas [10]. Such tools may be able to highlight areas where intensified control efforts would provide benefits without the significant resource requirements of expansive prevalence surveys with sophisticated diagnostics [11,12]. Recent efforts have been made to map the occurrence and endemicity of *T. solium* at the global and regional levels using the available literature on populations, disease occurrence, and risk factors [12,13,14,15]. Whilst this is critical work that highlights the scale of the disease on a broad scale, these efforts do not provide countries with detailed information that can aid current control efforts. At this point in time, studies on the spatial epidemiology of *T. solium* from academic institutes have primarily focussed on the observational occurrence and association of human and porcine cases [16,17,18,19,20,21]. To date, there have been no published efforts that have investigated the spatial autocorrelation of risk factors to identify high-risk areas. In situations in which disease occurrence data are lacking, the distribution of risk factors may act as an effective proxy to inform decision-making.

### 1.2. The Lao People’s Democratic Republic

The Lao People’s Democratic Republic (PDR) is the smallest and only landlocked country in Southeast Asia. It is a country with a diverse population that has seen conditions within the country change significantly since its establishment in 1975. Vast improvements to health care and standards of living have been made in recent decades; however, there is still significant variability across the country. Subsistence agriculture continues to employ over 60% of people in the country, and agricultural households are twice as likely to fall back into poverty [22]. Livestock is the second largest agricultural industry in the country, yet 95% of livestock owners are considered subsistence or smallholder farmers, with livestock sales contributing up to 50% of cash income in some northern districts [23]. Pig production is a central practice for these livestock households, particularly in the northern regions [24]. Pigs are a valuable source of income and have cultural and ceremonial significance in many communities [25]. The levels of sanitation and health provision in rural areas, combined with the nature of pig rearing and raw pork consumption, mean that *T. solium* is a substantial risk. Ensuring that subsistence and smallholder pigs can be raised safely and sustainably is critical for the livelihoods of much of the population in Lao PDR.

Multiple biological surveys investigating human taeniasis have been conducted in Lao PDR over the past 30 years. However, the diagnostic tests used in these surveys generally do not differentiate between different species of *Taenia* [26]. The government of Lao PDR has highlighted *T. solium* as a priority disease; however, further investigation into the epidemiology of *T. solium* in Lao PDR has been delayed due to the complexity and resources required for diagnosis. The use of spatial statistics has the potential to inform *T. solium* activities in Lao PDR, where larger structured surveys are not currently feasible.

Given the significance of *T. solium* as a neglected tropical disease and the ongoing calls for methods to identify hyperendemic areas, the objective of this study was to apply spatial autocorrelation statistics to readily available data to identify high-risk areas in Lao PDR. This objective aims to support decision-making for the control of *T. solium* in Lao PDR and provide an example of how such an approach may be applied in other endemic countries.

## 2. Materials and Methods

This study had four main methodological parts:

1. Obtain and handle risk factor data;
2. Apply global spatial autocorrelation statistics to determine whether spatial dependency was present for each risk factor on a national scale;
3. Identify which villages were considered hot spots for each risk factor using local indicators of spatial autocorrelation;
4. Assess the co-occurrence of risk factor hot spots and calculate a risk score for each village.

### 2.1. Data

Risk factor data from the Lao PDR 2011 Agricultural Census and 2015 Housing and Population Census were accessed via the Lao Decide Info platform [24]. Five key risk factors were selected for analysis on the basis of an unstructured literature review and expert opinion (Table 1). Risk factor data were standardised by the number of households in a village to control for the effect of village size. The two censuses were conflated so that risk factors could be considered in all villages. Polygon geometries from the 2015 census were considered the most representative of the current population. Consequently, point geometries of the 2011 villages were aggregated into the 2015 villages. After conflation, 185 of 8499 (2.2%) villages were missing agricultural data. Missing data were imputed using the mean of the district for the respective risk factor. All statistical analysis was completed using RStudio [27].

### 2.2. Global Spatial Autocorrelation

Global autocorrelation was assessed using global Moran’s I and global join count statistics [28]. Moran’s I statistic describes the similarity in values of a numerical variable between locations [29]. The relationship between locations is realised through a spatial matrix, where locations closer in space are weighted more heavily than those that are distant. Moran’s I generally ranges between +1 and −1, where a result of +1 represents perfect positive autocorrelation, and locations near each other have similar values. A result of −1 represents negative autocorrelation, and locations near each other have dissimilar values. A result of zero reflects no spatial autocorrelation, with values being randomly distributed. The significance of the statistic is inferred against the null hypothesis that no spatial autocorrelation is present and that values are randomly distributed.

Moran’s I is not an appropriate choice of test for categorical data, and global autocorrelation of binary risk factors was assessed using the global join count statistic [30]. The join count statistic involves counting the number of different joins between neighbouring locations. The joins considered were 1–1, 1–0, and 0–0. For the two binary risk factors of the main water or sanitation type, these values represented the following categorical joins between two neighbours: unimproved–unimproved (U–U), unimproved–improved (U–I), and improved–improved (I–I). If autocorrelation was present, then similar values were located closer to each other, and we would expect more U–U and I–I joins than I–U joins. The resulting I–I and U–U counts were significance-tested against their expected numbers if there was no autocorrelation, the null hypothesis. 

### 2.3. Local Spatial Autocorrelation

Global measures of autocorrelation are extremely useful in providing analysts with an overview of spatial dependency; however, these do not consider the potential for instability in dependency across an area. More detailed information on local spatial dependency is often required for decision-makers, particularly in resource-limited settings. Local Getis–Ord and local join count statistics were assessed as measures of local autocorrelation, with their significance tested using conditional random permutations [31]. Local Getis–Ord, or G, statistics examined each village and its neighbours, comparing their mean value to an expected value based on the global mean [32]. Positive results indicated clusters of values higher than the mean (hot spots), and negative results indicated clusters of low values (cold spots).

In a similar fashion, the local join count statistic was applied to binary variables [33]. Neighbouring joins of 1–1 (U–U) were counted for each location and tested for significance against the expected value given spatial independence. Due to the nature of the local statistic equation, any joins including zero (1–0 or 0–0) multiplied to zero and were not considered. As a result, the statistic was only of use in locations with a value of one (i.e., hot spots). The categorisation of the one and zero binary values could simply be reversed to assess hot spots of the second category, for example, improved sanitation or water rather than the unimproved category, if necessary.

### 2.4. Village Risk Scores 

The ability to examine a single risk metric is extremely useful for decision-makers. To accommodate this, each village was provided with a risk score based on the count of risk factors that was classified as a hot spot in that village. Due to the importance of pig households in the epidemiology of *T. solium*, this calculation was repeated and limited to villages that were pig hot spots.

## 3. Results

**Figure 1:**
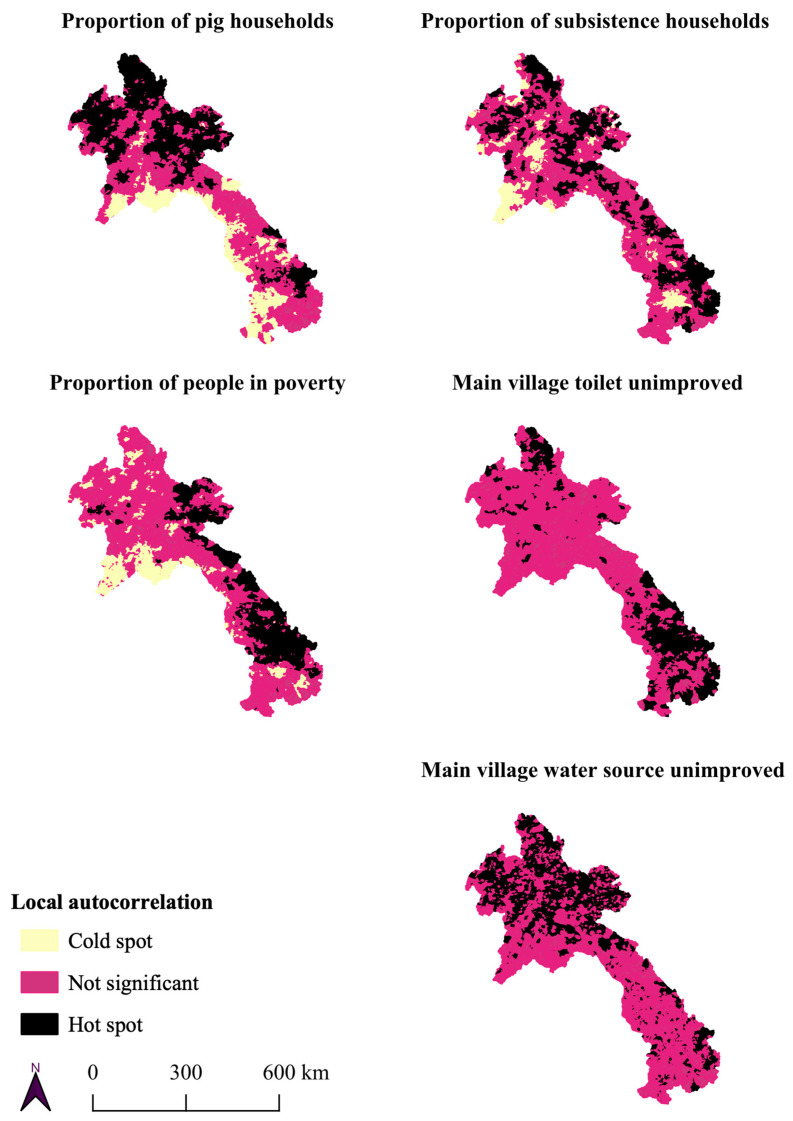
Mapping of local autocorrelation statistics.

All risk factors related to *T. solium* in Lao PDR were spatially dependent in Lao PDR. Moran’s I statistic was positive and significant across five orders of spatial lag. The results ranged from 0.74–0.51, 0.71–0.43, and 0.59–0.25 for pig households, poverty, and subsistence households, respectively (Supplementary File S1). The binary risk factors of the main sanitation or the main water source being unimproved also demonstrated spatial dependency (Supplementary File S1). Local autocorrelation statistics illustrated that this dependency was not homogenous, with 15–26% of villages being identified as hot spots depending on the risk factor (Table 2). The locations of the risk factor hot spots illustrated that northern Lao PDR appears to have a larger number of hot spots of pig households and unimproved water. Meanwhile, hot spots of subsistence households were more evenly distributed. The majority of poverty and unimproved sanitation hotpots were found in southern Lao PDR; however, there were hot spots for all risk factors scattered throughout the country (Figure 1).

**Figure 2:**
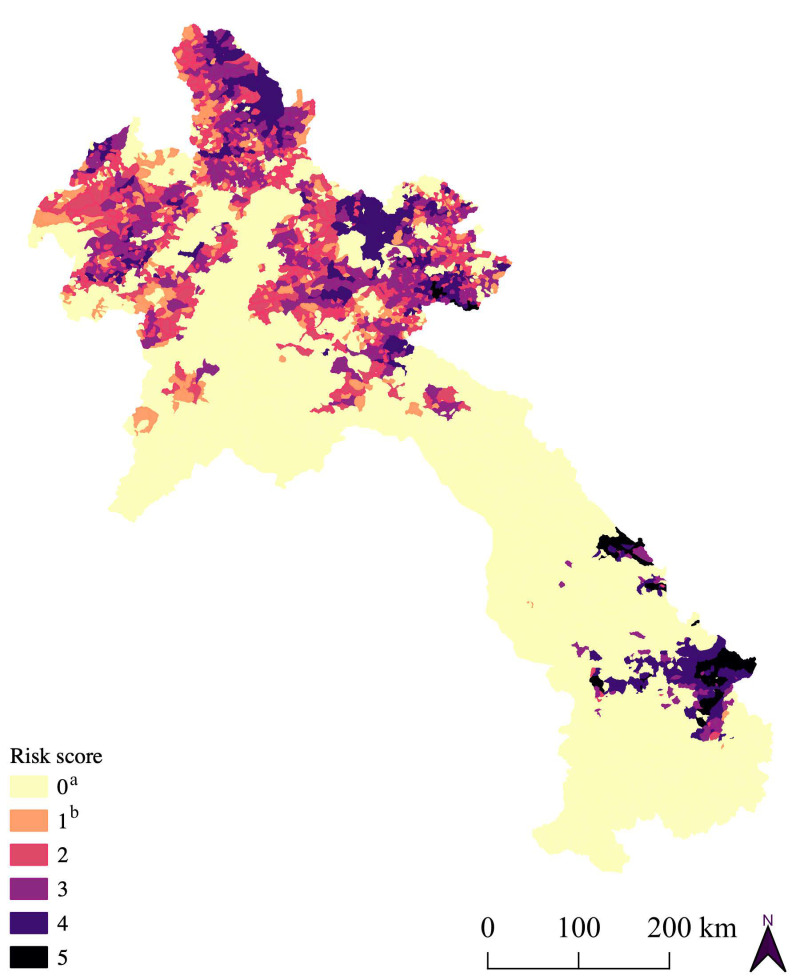
Mapping of local autocorrelation statistics. ^a^ Not a pig hot spot. ^b^ Pig hot spot only.

The co-occurrence of risk factor hot spots was present in approximately one in three villages (31%) (Table 3). This percentage decreased when considering only pig hot spots and the co-occurrence of other risk factors. One in five villages (20%) was classified as a hot spot for pigs and another risk factor (Table 3). Mapping these villages revealed northern Lao PDR as the largest area with overlapping hot spots for pigs and other risk factors. However, there was a smaller area in southern Lao PDR where villages were considered hot spots for almost all risk factors (Figure 2).

## 4. Discussion

Passive reporting, anecdotal knowledge, and limited surveys all support the major finding that northern Lao PDR is the largest area of high risk for *T. solium* in Lao PDR [9,34,35,36,37]. The identification of a second high-risk area in southern Lao PDR is of interest, as there has been little investigation in this location. It is hoped that these results can encourage further research and targeted investigation where appropriate. To date, only a single hyperendemic village has been documented in Lao PDR [26]. The village of Om Phalong in the northern province of Phongsaly was identified in 2013 [35]. The subsequent trial of porcine and human interventions in a One Health manner led to the cost-effective control of *T. solium* and soil-transmitted helminths in the village [38]. This trial provided some of the first empirical evidence for how health can be enhanced through a One Health approach in Southeast Asia; however, the village has now reverted to a hyperendemic state five years later (unpublished data). A wider body of evidence for cost-effective interventions for the control of *T. solium* is currently lacking and has been identified by experts as a critical research area [39]. The results presented above classified this hyperendemic village as a hot spot for pig, subsistence, and water risk factors and revealed other areas that are high-risk and may include similar villages.

This study presents a tool that may allow endemic countries to take the first steps in planning for the eventual control of *T. solium* by identifying high-risk areas that should be prioritised for further investigation. The recently released WHO risk-mapping tool [40] uses a semi-quantitative method to combine binary data on porcine cysticercosis, *T. solium* taeniasis, unknown taeniasis, neurocysticercosis, open defaecation, and backyard pigs to produce a risk level for a given area. This method is heavily reliant on disease occurrence data, with four of six factors focussed on this, and it only considers binary risk factors. Relying on disease occurrence data means accepting the limitations of suboptimal *T. solium* diagnostics and the health systems needed to capture, analyse, and store such data. These limitations have been noted in regional attempts to map the risk of *T. solium* [13,14,15]. The use of pre-existing census data highlights the utility of what already exists, and it is hoped that this tool can be applied in other endemic countries. The use of readily available non-biological data is particularly relevant in Lao PDR, where health and veterinary services are limited. Public spending remains low in both sectors, with a reliance on out-of-pocket spending and external funding. This context means that the efficient identification or screening of hyperendemic villages is increasingly important.

The use of open-source software and versatile methods was an important feature of this study. The analysis can be completed by national staff on software that only requires basic training or extension. This kind of analysis and training on existing data is critical to capacity development and the next users who may wish to repeat or alter the analytic method in the future or apply similar approaches to other issues or locations. Although the national results presented provide an indication of the high-risk areas, as more is learnt about the occurrence and associations of *T. solium* in Lao PDR, repeated spatial analysis on future census data and at a finer scale has the potential to reveal further local spatial patterns that may be hidden in this study and prove critical to local control.

This study considered a small number of risk factors on the basis of their availability in census data, an informal search of the literature, and expert opinion. Other risk factors, such as those relating to culture and ethnicity, were considered relevant and potentially important; however, their data were not available, and evidence of the association between these factors and *T. solium* in Lao PDR is limited. This sparsity of data is also the reason why this risk mapping tool has not yet been validated against biological data. Due to the diagnostic challenges previously mentioned, there are insufficient data at the village level to appropriately validate this tool [26]. This lack of data also prevented the application of more sophisticated risk mapping methods that use prevalence as the outcome or dependent variable. As more prevalence data become available in Lao PDR, such an approach is encouraged. This issue of data sparsity is common across many endemic countries; it is why a non-biological risk mapping approach was taken in this case, and it is believed to be relevant to the many endemic countries that are trying to better understand their own risk patterns of *T. solium.*

Further validation of this tool is necessary because whilst the hyperendemic village mentioned above was correctly identified by the tool, the only village where there was a published case of neurocysticercosis [37] was not identified as a hotspot for any risk factors. It is hoped that this tool can be validated in a stepwise manner as *T. solium* research and control progress in Lao PDR. There is also the potential to apply species-specific molecular diagnostics to taeniasis-positive patients in national helminth surveys, which may provide a suitable validation dataset and rapidly progress the understanding of all *Taenia* species in Lao PDR. This includes *T. asiatica*, which shares similar biological features to *T. solium* and may be present in similar high-risk locations; however, the current epidemiology and significance of *T. asiatica* remain unknown in Lao PDR, as it has only been identified in three cases [26]. The tool is not presented here and should not yet be interpreted as a complete predictor for disease occurrence. Rather, it is a risk mapping and decision support tool that helps to identify high-risk locations for further investigation, analysis, and ground truthing. The tool should be used in combination with passive surveillance systems, existing knowledge, and other methods prior to significant resource allocation.

This study has four other limitations that are worthy of discussion. Firstly, the method for hot spot classification is data-driven and based on first-degree neighbours. Consequently, it does not consider biological or epidemiological thresholds that may be linked to disease occurrence. This is simply because these thresholds are currently unknown in Lao PDR. Secondly, the nature of local autocorrelation statistics means that a village’s hot spot status is impacted by its neighbours’ values. There is the possibility that a single outlier village may have its value averaged out by its neighbours, resulting in it potentially not being classified as a cluster. Whilst described elsewhere as a significant limitation [41], the purpose of this analysis was to identify broad areas for further investigation. The inclusion of neighbouring values may begin to account for porous village borders and the fluid nature of many communities. As such, the use of first-degree neighbours may be a limitation or a strength of this approach. This will not become clear until there are further studies related to the village-level occurrence of *T. solium* in Lao PDR and elsewhere. Thirdly, the strong global autocorrelation that is present in the national data may make the detection of local clusters more difficult and hide distinct local patterns [32]. This limitation could potentially be overcome by repeating the analysis on smaller scales, focusing on the now-identified high-risk areas. This limitation highlights the importance of simple and versatile methods that can be reapplied readily as new information comes to hand. Finally, when examining the co-occurrence of hot spots, it should be remembered that these results only reflect co-occurrence and, importantly, do not consider the correlation, interaction, or relative weight of each risk factor. Further investigation of combined risk could be achieved through multi-criteria decision analysis or other methods. This study is a starting point in the investigation of *T. solium* risk in Lao PDR; however, these limitations are a reminder that multiple lenses should be applied in health planning prior to decision-making. Assumptions and analyses should be regularly re-examined as new data and information become available. For this reason, future risk mapping tools for *T. solium* should ideally continue to be simple, rapid, and versatile.

## 5. Conclusions

This study applied simple spatial autocorrelation statistics to pre-existing data and produced results that are relevant to local stakeholders and illustrate the spatial heterogeneity of *T. solium* risk factors at a sub-national level. The information provided by this study allows for the targeted application of further research and control activities in Lao PDR in a clear and transparent manner. Investigating the spatial clustering of risk factors offers a simple, versatile, and rapid approach that can assist decision makers in identifying high-risk areas. This approach is pertinent to *T. solium* due to the current lack of accurate, reliable, and practical diagnostic tests. Further research and significant investment into the fundamental epidemiology of *T. solium* and options for its control are required in Southeast Asia if the disease situation is to be better understood. Given the intrinsic link between living conditions and other neglected tropical diseases, the approach presented here has the potential to inform control efforts for other neglected tropical diseases in Lao PDR and other countries.

## Figures and Tables

**Table 1:** Risk factor data.

Risk Factor	Data Type	Source
Pig-owning households	Proportion in village	2011 Agricultural Census
Subsistence households	Proportion in village	
People living in poverty	Proportion in village	2015 Housing and Population Census
Main sanitation unimproved	Binary	
Main water source unimproved	Binary	

**Table 2:** Summary of local autocorrelation statistics with 95% significance.

Risk Factor	Number of Villages (% of Total)		
	Cold Spot	Not Significant	Hot Spot
Pig households	2413 (28%)	3866 (46%)	2220 (26%)
Subsistence households	1494 (18%)	5113 (60%)	1892 (22%)
Poverty	1897 (22%)	5120 (60%)	1482 (17%)
Main sanitation type	n.a. ^1^	7219 (85%)	1280 (15%)
Main water source	n.a. ^1^	6796 (80%)	1703 (20%)

^1^ See interpretation of binary local join count statistics described in methods.

**Table 3:** Summary of village risk scores.

Risk Score	Number of Villages (% of Total)			
	Any Risk Factor		Pig Hot Spot	
0	4192	(49.3%)	6279	(73.9%) ^a^
1	1663	(19.6%)	480	(5.7%) ^b^
2	1439	(16.9%)	802	(9.4%)
3	831	(9.8%)	595	(7.0%)
4	327	(3.9%)	296	(3.5%)
5	47	(0.5%)	47	(0.6%)
Total	8499	(100%)	8499	(100%)

^a^ Not a pig hot spot. ^b^ Pig hot spot only.

## Data Availability

All data generated or analysed during this study are included in this published article (and its Supplementary Information Files).

## Supplementary Materials

The following supporting information can be downloaded at: https://www.mdpi.com/article/10.3390/tropicalmed8040221/s1, Supplementary File S1: Global autocorrelation results.
