# Supplementary material for: A Spatial Autocorrelation Method for Taenia solium Risk Mapping: The Case of Lao PDR
Source: Trop Med Infect Dis. 2023 Apr 10;8(4):221. doi: 10.3390/tropicalmed8040221 (PMC10146971; doi:10.3390/tropicalmed8040221)
Supplement: Supplementary file 1 [file tropicalmed-08-00221-s001.zip › tropicalmed-2309757-supplementary.pdf]

Figure S1. Global Moran's I statistic across five spatial lag orders.

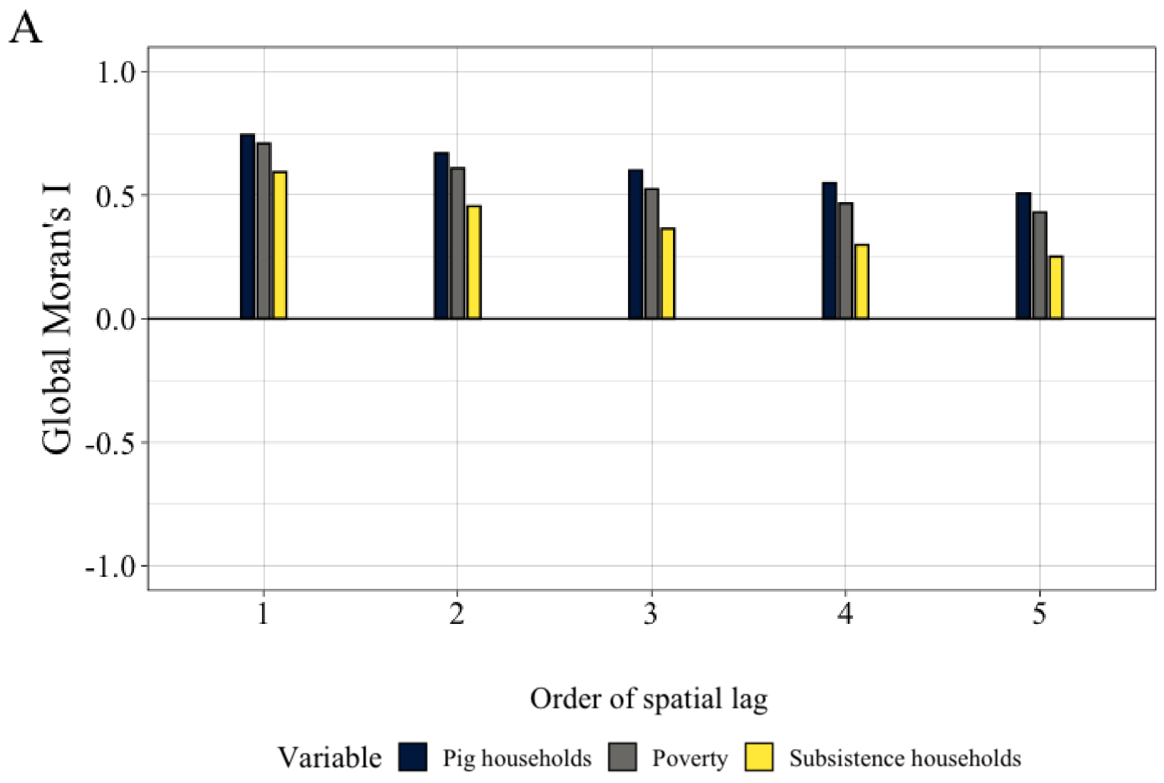

Table S1. Global join-count statistics for binary variables

**Risk factor**	**Global join count statistic**		
	**Expected**	**Result**	**p-value**
Main sanitation type			
Improved-Improved	10,641	13,108	<0.001
Unimproved-Unimproved	2,899	5,587	<0.001
Main water source			
Improved-Improved	4,837	8,148	<0.001
Unimproved-Unimproved	7,647	11,220	<0.001
